# The Martyrology of Medicine

**Published:** 1909-08-28

**Authors:** 


The Hospital

A Journal of

The Medical Sciences and Hospital Administration.

New Series. No. 131, Vol. V. [No. 1203, Vol. XLVI.] SATURDAY, AUGUST 28, 1909.

### THE MARTYROLOGY OF MEDICINE.

Long ago, when medicine was a mystery even to the adept, Tertullian crystallised a truth into an epigram when he stated that "the blood of the martyrs is the seed of the faith." Wise and thinking men, like the Emperors Julian and Akbar and the Chancellor Gerson, have recognised the importance of that truth in matters of religion, and generally in matters of philosophy, art, or science, the great leaders have been at one with them in admitting the futility of persecution as an argument of persuasion. Nevertheless there have been times in the history of art, philosophy, and science, no less than in the history of religion, when the dogmatist triumphed over the patient believer and when to persecute was deemed better than to investigate. The story of such occasions must ever be a sad and dismal one to read. We are tolerant to-day, and toleration, it is satisfactory to note, is getting a more powerful factor in the ethics of civilisation as men get more enlightened. And yet there are tales of martyrdom which are not as old as the Koran, and of persecution much nearer our own day than the times of Calvin and Torquemada. To those of us who are under the impression that scientists are more broad-minded, more liberal, less intolerant than philosophers, artists, or theologians, a glimpse into the martyrology of medicine will prove elucidative, though at the same time painful and intensely shocking to the supreme self-satisfaction that many of us feel in thanking Providence that we are not as other men are.

Sir William Sinclair has therefore done well to put before English readers a clear study of the life and history of one of the most interesting medical martyrs of modern times.* The martyrdom of Semmelweis was not indeed one that can compare in physical severity with that of Servetus or Damian, but it was none the less a martyrdom. Had men like Klein, Braun, and Scanzoni possessed the power or the means to lead the patient young Hungarian investigator to the scaffold, we may be assured that the discoverer and elucidator of the ætiology of puerperal fever would never have gained his professorship at Pest. Fortunately scientists have never held the power that was accorded to theologians, and Semmelweis died a natural death—dying, as Von Waldheim remarks, a victim to pyæmia, the disease whcse identity with puerperal fever he had been the first to recognise and to the prevention of which in midwifery, gynæcology, and surgery, he had devoted his energies as a teacher. But before his death, for nearly a quarter of a century, he was made to suffer for his temerity in daring to dispute the then dogmas of medicine. Nowadays the tnird-year student laughs with impunity at these dogmas; the heresy of to-day (to quote a Church Father once more) is the creed of to-morrow. Semmelweis's heresy has become the faith of the profession, and his detractors and persecutors, with few exceptions (and such only those who, like Virchow and Simpson, whole-heartedly confessed their errors and became supporters of the new theory) live, with the critics of Keats, each but as a

Noteless blot on a remembered name.

Medicine has ultimately acknowledged the just claims of the pioneer, but the long, heartbreaking struggle which rewarded the brilliant inspiration and the patient research was a veritable Progress towards Calvary for Semmelweis. It was a martyrdom, and, as has been so often pointed out, one of the most indefensible of all in the martyrology of medicine. Here and there a few bright points flicker in its sombreness. For instance, from the wearying attacks of his German colleagues, it is refreshing to turn to the recital of one Englishman's support. The late Dr. Routh, who introduced the new theory to the notice of the London obstetricians, remained an enthusiastic convert, and loyally aided the spread of the new doctrines in this country. Much less gratifying to English readers is the story of the resistance of illogical dogmatists of the type of Denman, whose antagonism considerably retarded the work of progress.

There is a lesson in all this for every professional man to-day. It is the lesson of that supreme tolerance which some of us are inclined to admit in principle and flout in practice. In medicine, as in all other professions, there is a tendency towards trade unionism, but there is a further tendency, which it shares solely with theological sects—namely, to

* "Semmelweis: His Life and His Doctrine." By Sir Wm. Sinclair, M.D. Manchester University Series.

insist increasingly on the weight of authority and dogma. Thinking men will readily agree that such tendencies are not in the best interests either of the profession as a whole or of medicine as a science. Our highest aim is to investigate truth, not to buttress theories by the weight of unassailable authority, and it would be a sad time for medicine when it is decreed that an associational meeting is as infallible as an ecumenical council. The martyrology of our profession is large enough already; there is no room for further indiscretions in the exercise of the privilege of expert criticism.

